# Sport-Induced Substance Use—An Empirical Study to the Extent within a German Sports Association

**DOI:** 10.1371/journal.pone.0165103

**Published:** 2016-10-26

**Authors:** Monika Frenger, Werner Pitsch, Eike Emrich

**Affiliations:** Department Economics and Sociology of Sport, Faculty of Human Sciences and Economics, Saarland University, Saarbrücken, Germany; University of Rome, ITALY

## Abstract

In cooperation with the Sports Association of the Palatinate (SBP), a survey was conducted on substance use by recreational and amateur athletes. Distribution of the online questionnaire took place by means of chain-referral sampling, and questions on substance use were presented using the randomized response technique (RRT) to protect the anonymity of respondents and prevent socially desirable answers. The estimated lowest limit for the population share for use of prohibited substances during the last season (4%) and for lifetime use (3.6%) did not differ significantly. Approximately 21% of respondents had used substances for training or competitions that were taken for a purpose other than performance enhancement (e.g., to improve their mood or to help with recuperation from a minor injury or illness) in the last year. 49% had done so at some point in their life.

## Introduction

In recent years, there has been a multitude of studies on doping in recreational and in amateur sport. As a result, the extent of this phenomenon was clarified for special populations (like e.g. bodybuilders [1–2]) and for special substances (mostly for anabolic steroids [3–9]). Nevertheless, the prevalence in the total population of amateur and recreational sportsmen and -women remained unclear due to several analytical, methodological, and empirical issues. Despite the uncertainty of prevalence data, doping in recreational and in amateur sport has been labelled a public health issue [10–12]. The following article aims to supplement this argument with a sound estimation of the prevalence of doping in a general population of sportsmen and -women.

## Theoretical Assessment of the Problem Area

### State of research

It is immediately evident that the large number of studies on the prevalence of doping in recreational and amateur sport taking into consideration only certain substance classes or special sport populations can only provide limited estimates of the prevalence within the total population. Therefore, we will first focus on studies that address either unspecific or general substance (ab-)use, or that address general populations with only minor limitations in socioeconomic parameters, especially those studies without limitations by sport discipline.

A survey among students at four universities in Belgium, Demark, Germany, and Switzerland (population: well educated young European adults) on the use of enhancing substances for both mental performance (neuro-enhancement) and sports performance (doping) with the Randomized Response Technique (RRT) (see methods section in this article) showed that the prevalence of doping in that population was 5%, while the prevalence of (neuro-) enhancement ranged between 3 and 8% [13]. It should be noted here that this proportion only represented the reliably estimated lower boundary of “honest yes respondents” in the Randomized Response estimate (see methods section). The existence of respondents who failed to follow survey instructions may have led to an inaccurate estimate of the potential number of users. Another study, conducted at the German Sport University Cologne, revealed a higher prevalence of substance use at 11.2% among students (population: young, well educated German adults, highly interested in sports). This figure resulted from analyses of urine test samples and is not affected by any voluntary bias. However, the prevalence rate may be elevated due to the specific nature of the university, at which only sports students were questioned [14].

Concerning the substance use of young male adults in school and university environments, unspecified substance use has also been studied using direct questioning. With a wide definition of performance enhancing substances, which also include dietary supplements, the prevalence was found to be about 31% [15]. This rate suggested a high level of readiness to enhance performance among the sample. Among those who indicated they had used performance enhancing substances, 31% confessed to having used illicit substances according to the NCAA guidelines. This resulted in a doping prevalence of 9.6% for this population. For a similar population from Sweden, the overall proportion of doping was reported at 1.6% [16]. A study among Italian high school attendees found a prevalence rate of 1.5% for using illegal performance enhancing substances, while the total rate of substance use for performance enhancement was 6.8% [17]. As the focus of the study was substance use during the last three months, the reported prevalence might be low in comparison to other studies considering a longer period for reporting use.

To critically assess the evidence from these studies, it is important to note that the first two studies used methods which were either impervious to social desirability bias [14] or that were designed to minimize it [13]. This was not the case for the latter studies that were conducted using direct questioning. As a result of these survey findings on relatively large populations for general substance use to enhance physical performance, we estimated the overall prevalence between approximately 5% and 10% in the total population.

Now we turn to studies that additionally identified risk factors for smaller populations or for single substances or substance groups. In order to structure the large number of studies, we will categorize the discussion based on the different determinants studied.

The most important and highly stable social background variable is gender, with female respondents generally showing a lower propensity to dope. Its significance as a determinant of doping prevalence in amateur and recreational sport was shown in studies among gym users [1–2, 18] as well as for the use of anabolic androgenic steroids among university students ([3–9, 16], 14 more studies from the United States, cited in [5], 25 studies and three periodically conducted national youth surveys in the U.S., cited in [11], and three studies from Great Britain and Germany, cited in [10]). This effect was also shown in a special mixed sample of at-risk individuals [9], as well as in studies on doping substances in general among university students [17]. Few studies where the influence of gender was investigated offered no support for this determinant [19] or provided contradictory results [20]. Although this pattern is stable, there have been only few attempts to link these results to theoretical issues such as gender specific health-related behaviour or gender specific risk behaviour.

Additionally studies have identified further social background variables such as social status [4, 7, 9, 12, 20, 21] and the intensity of involvement in sport [2, 7, 17, 19, 22] as determinants of doping. As we were not able to test hypotheses on these parameters due to the limited number of responses to our survey (see results section), we will not discuss these variables in detail.

Besides these social background variables, an increased prevalence among users of other legal or illegal substances has been reported in several studies [2, 6–9, 15–16, 22–24], though there were some inconsistent results concerning various drugs [22].

Drawing on this research, we hypothesized that doping prevalence in recreational and in amateur sport differs by gender. In general, this study did not aim to identify new determinants for doping, but to 1.) estimate the overall prevalence, and 2.) to determine if gender is a determinant for doping. So far, gender has only been shown as a determinate in limited populations and mostly for single substances.

### The concept of doping in recreational and amateur sport

The concept of doping has evolved in the context of professional elite sport and it is unclear if this concept can be adopted in sporting activities outside the professional realm. It is important to note that the definition of substance use relevant to this study is not necessarily that promulgated by the World Anti-Doping Agency (WADA). To clarify the concept of “substance use in recreational and amateur sports”, we must differentiate between three definitions.

1. Substance use, or doping, according to the World Anti-Doping Code (WADC): This code defines doping in terms of a list of prohibited substances or methods. Whether or why the athlete intends to use these substances or methods does not matter, due to the strict liability principle. Additionally, under this definition doping does not require intent to enhance one's performance, because there are additional reasons why substances are prohibited (e.g., the “spirit of sport” argument).
2. The European Union’s (quoted in [25]) definition of substance use: The core concept is the intention to unfairly enhance one's athletic performance; the specific techniques and methods used therefore do not matter at all.
3. Lay persons' understanding of substance use in sports contexts, which can be derived, for example, from discussions on the internet: In this context, neither the intention to increase one's performance nor a specific list of substances matter. For example, some pain-killers are discussed as “doping substances”, while pharmaceuticals that are included on lists of banned substances are not necessarily seen as doping as long as they are used for (self-) therapy.

If participants in mass sport events are members of a sports club, they accept the rules of the WADC and of the sports organization, especially those of fair play, with their membership. Athletes in recreational and amateur sport, too, are expected to perform at their best by using only their talent and by training hard, even if there is no organized surveillance for doping violations. Doping surveillance and sanctioning can, at least in principle, be applied to those who participate in organized competitions. However, as there are typically no doping tests at the amateur level of sport there is no practical need for participants to be informed about the legal meaning of doping (WADA’s definition) or the list of prohibited substances.

Therefore, substance use in recreational and amateur sport is better understood in the sense of the Thomas theorem: “If people think that something is real, it is real in its consequences” [26]. In recreational and amateur sport, the lack of doping tests and their results mean that there is no institutional agent for defining behaviours as doping. This means the labelling of a behaviour by the participants in this field is most relevant, whether or not tests have been conducted. If the behaviour is labelled as doping, consequences such as loss of reputation and social marginalization are equally effective sanctions, regardless of whether the behaviour was doping in a legal sense.

In recreational and amateur sport, it is also important to consider the intent behind substance use. For athletes who are under surveillance in the WADA Anti-Doping Testing regime, the relevant question is only whether or not a substance is in the athlete’s body; it does not matter why this substance has been used. Applying this standard to recreational and amateur sport could have major consequences for the use of over-the-counter medicine. Taking a pill to treat nasal congestion caused by a cold may constitute an anti-doping rule violation if the pill contains pseudoephedrine hydrochloride. Under WADA’s definition, this is a prohibited substance. However, using it for self-treatment in a non-sports-related context would hardly be considered doping (see [27] for the differentiation between doping/enhancement and treatment in the general public). Using a medication to facilitate training participation when an athlete feels like he may have caught a cold might not be labelled prohibited substance use by athletes in recreational and amateur sport. On the other hand, using creatine, caffeine, or l-carnithin (none of which are prohibited) in marathon training [28] or using pain-killers in sport [29] has already been colloquially labelled doping by some in these populations.

Taking these observations together, substance use in recreational and amateur sport is understood by participants in this field as a behaviour

1. that is regarded as performance-enhancing, and
2. that is (self-) perceived as illegal.

Therefore, measuring prohibited substance use prevalence in this sense requires measuring the proportion of athletes who believe they have done something prohibited. This prevalence is an indicator of an openness to transgress between substance categories (i.e., from acceptable to prohibited) that is accepting of doping. Cases where prohibited substance use acceptance exists but has not (yet) led to substance use behaviour due to the absence of necessity or opportunity are known systematic measurement errors, which lead to an underestimation of the prevalence of a doping-tolerant mind-set.

## Method

### Study’s definitions–doping and sport induced self-medication

The operationalization of substance use has one important implication when measuring its prevalence: to rule out other reasons for substance use. When measuring the concept of “doping”, one has to emphasize three essential elements of our definition: “athlete”, “prohibited substances or methods”, and “the intention to enhance your performance in sport”. Our questionnaire asked explicitly about the use of substances that the respondent believes to be prohibited. Therefore, using substances that are not known to be prohibited would lead to the answer “no”, while substances that are thought to be prohibited would lead to the answer “yes”, irrespective of whether these substances are, in fact, prohibited. The defining element “athlete” ensures that respondents will refer to a behaviour that they perceive as related to their sports participation. Substances used for marginal health problems, which are not part of the social phenomenon “illicit substance use in recreational and amateur sports”, are beyond the scope of this definition.

In addition to these performance related behaviour, we were interested in the substance use for purposes other than performance enhancement. The differences to these two behaviours are 1) the intention (other than performance enhancement, such as pain reduction, mood, etc.), 2) that the reference to a specific sport is only relevant for doping but not for sport induced self-medication, and 3) that the substances which are used for doping are seen as illicit by the respondents, while in the case of self-medication this issue is irrelevant. By taking the substance, the athletes could, for example, try to reduce pain, accelerate recuperation, or improve their mood. Although any of these attempts can be seen as (also) performance enhancing, the main difference to doping is that medication use was not primarily intended to enhance performance.

We asked the entire sample about substance use for sport-induced self-medication (in tables and figure, we will use the abbreviation medi) over their whole sport life and the persons who were still active in the last year. This means we only asked about substance use linked to active sports participation. The original question (translated into English) is: “Have you ever (last year) used substances in training or competition which were not intended to enhance your performance?”

### Instruments (structure of the questions)

We used an online survey to investigate the prevalence of doping and sport induced self-medication. At the beginning of the survey, respondents were asked if they participate in sports and which sport disciplines they practice or have practiced. For these disciplines, we also asked if they participate/have participated in competitions. If the response was yes, we asked for their last competitive level, as well as for the highest competitive level they have ever reached. This was used to prioritise sport disciplines in our questionnaire (see below). These questions were followed by at least two and up to six questions concerning their sports-related substance use (see RRT procedure section). The questionnaire finished with socio-demographic information (i.e., age, gender, education level).

### Specific instrument—The RRT questions

**Fig 1:**
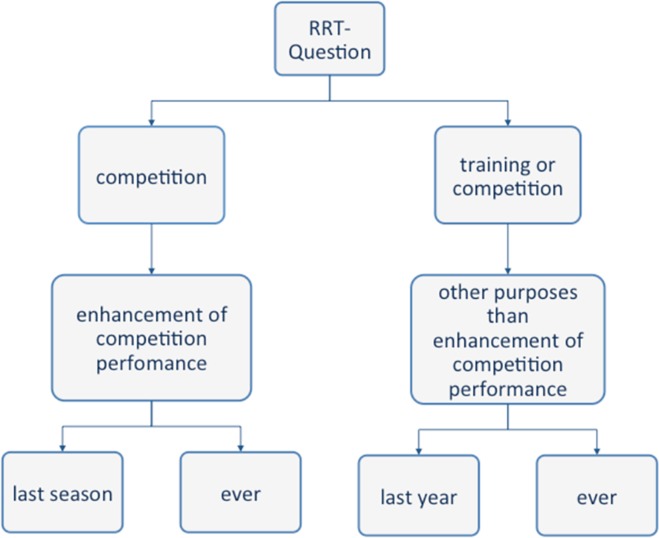
Overview of the questions about the embarrassing characteristic substance use that are asked using the RRT.

With RRT, we asked a maximum of two questions relating to doping (lifetime and last season) and two questions relating to medication (lifetime and last year). Because of that all participants got one question (sport-induced self-medication, lifetime) in minimum and could get up to four RRT questions. A summary of the questions surveyed using the RRT is shown in Fig 1.

The questions related to doping in the last season in one sport (basketball in our example, for the lifetime prevalence the question was modified accordingly) were asked in the following way: “Have you, as a basketball player, last season used prohibited substances or methods with the aim to enhance your performance?” This question was asked to those persons who participated in sport competitions over the previous season (or ever). The relevant sport was decided after the prioritisation (see instruments section).

In addition to the doping questions, we asked about sport-induced self-medication during the last year and over the respondent’s lifetime. For this question, we asked about use in the last year because non-competitive athletes were also questioned here and they might not find the term “season” helpful or intuitive, which may lead to respondents assuming different windows of time. These questions about sport-induced self-medication were not specific to any one type of sport, but according to the differentiation between doping and sport induced self-medication (see study’s definiton section), they explicitly excluded the intention to enhance sporting performance.

### The RRT procedure

Because admitting to doping can be highly embarrassing, we used the randomized response technique (RRT) for questions on doping and medication. In addition to ensuring our results are comparable to other surveys [13, 19, 30–33], this approach leads to more reliable answers than those obtained by asking direct questions because RRT reduces distorting influences, such as those resulting from social desirability [34–35]. RRT cannot erase all social desirability bias, but in our opinion it guards against the worst fear, which is of exposure. We know from methodological studies on RRT [36–41] that results come closer to capturing true prevalence than direct questions. We only know two studies with lower prevalence estimations in the RRT questionnaire than when questioning directly [42–43]. That may be the result of reduced fear, since RRT questions can moderate the weight of expectation to answer “correctly” by means of social desirability. Additionally, recent publications have shown this technique produces reliable estimations of doping prevalence in sport (see [30–32] for elite sport as well as [13, 19, 33, 44], for sport below the professional realm).

This method protects against bias by giving an additional instruction when answering a question for an embarrassing property. Depending on which result is randomly generated from a known distribution, respondents either answer the embarrassing question or a corresponding innocuous question. In our case, this second question is structured in such a way that a cooperative person will always answer it with “yes”.

The literature contains reports on the use of different random generators [45–47] or other randomly generated characteristics such as the final digit of telephone numbers [48–49]. However, for an Internet survey, it cannot be assumed that all respondents will have easy access to a random generator in the form of a coin or die precisely at the moment they are taking part in the survey. Other randomly generated characteristics, such as the final digit of a telephone number, assume the researchers know the distribution of telephone numbers at the moment the survey is administered. In this survey, we decided to use randomly generated numbers with equally distributed digits.

**Fig 2:**
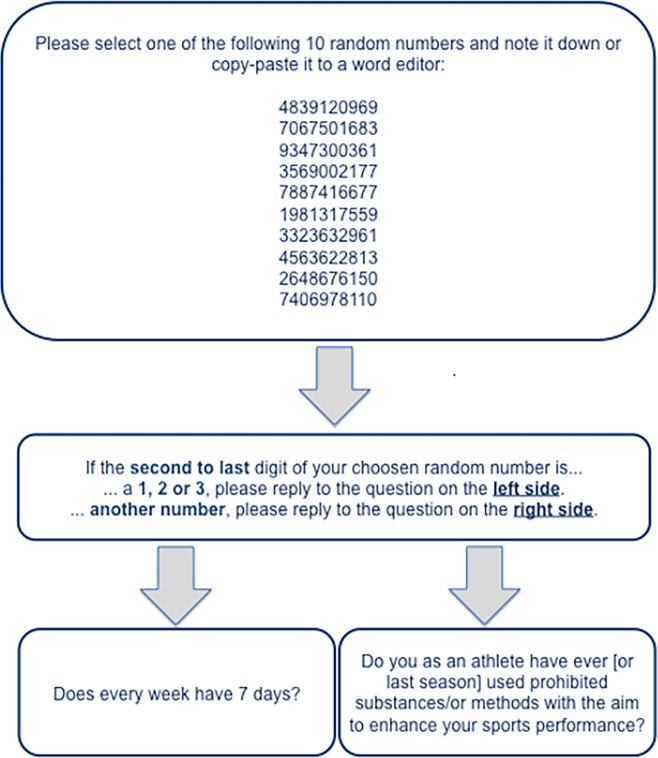
Example of an RRT question similar to those asked from respondents.

The process is illustrated by means of an example in Fig 2 that shows the forced answer model of the RRT for qualitative characteristics [50] (for other variants, such as the unrelated question model or for quantitative characteristics, see, for example, the summary in [45]).

Because the researcher does not know the random number generated for the respondent, he cannot conclude from a “yes” answer that the respondent has actually used prohibited substances or methods. However, because we do know the distribution from which the random number is generated, we can derive the probability that the respondent is instructed to answer the embarrassing question. From this, the proportion of people in the population exhibiting the characteristic (here, athletes who have taken substances) can be calculated.

Despite special instructions, cases continue to occur in which, for unknown reasons, the respondents do not comply with the RRT procedure [51–52]. These cases of so-called “cheating” can occur for a variety of reasons (for example, deliberately not complying with the instruction, not understanding the instruction, or similar errors) and thus reduce the precision of the estimate. To control for these biases, the “cheater detection model” [53] has been developed. The cheater detection is based on the assumption that RRT estimates of population shares are independent from the probability to answer one of the innocuous questions or the embarrassing question. To detect cheating, the sample is randomly split into (normally equally sized) subsamples with different probabilities. With these two groups, we can estimate three population proportions, namely the rate of honest yes responders, the rate of honest no responders and the rate of respondents who do not answer according to the instructions. In the literature on RRT, this third proportion is called “cheaters” although this does not imply that these respondents did not follow the instructions deliberately, nor that, in the present case, they cheated in the sense that they used prohibited substances. Nevertheless, we will use the term “cheater” for this population share according to the terminology in this special field of statistical methods. Further analysis of the RRT method with cheater detection is available elsewhere [54].

### Data Analysis

The RRT can be used for exploratory prevalence estimation as well as for hypothesis testing, although the statistics needed for this second purpose are not straightforward. For the variants of RRT used here, error components are typically not distributed normally, but are heavily skewed [13]. Therefore, significance tests should be performed using bootstrap methods to calculate confidence intervals independently from any distribution assumption [55–56]. When using bootstrap statistics the meaning of a significant difference between two groups must be reinterpreted. For our purposes, the difference between the two groups under study and the (bootstrapped) confidence interval of the difference are used, and the reference bcα-(bias corrected and accelerated) value is reported in Tables A-D in S1 Appendix (5^th^ column). For two groups to differ significantly, the value of the difference and the limit both must be positive or negative. Otherwise, the test fails and the hypothesis is rejected.

With this method, results of statistical tests are reported in a distinctive way because it lacks the value of a statistic (such as a t-value or an F-value from an ANOVA) or degrees of freedom. Additionally, the often-reported p-value, indicating the estimated significance level, becomes meaningless. A p-value could be reported [55], but would likely vary between different bootstrap-simulations even if the null hypothesis were consistently rejected at the selected level for significance (here: p<0.05). The results of the bootstrap analyses are shown in the additional tables (Tables A-D in S1 Appendix).

### Ethical Issues

According to the guidelines of the German Research Association, no ethical approval was needed because the research did not pose any threats or risks to the respondents and the respondents were fully informed about the objectives of the study (http://www.dfg.de/foerderung/faq/geistes_sozialwissenschaften/index.html). A member of the ethical committee of Saarland University confirmed that ethical approval was not needed for this study.

Conformity with the data privacy act of the European Union is confirmed by the data security officer at Sportbund Pfalz. This includes that neither participation nor non-participation could render negative consequences to the addressees, and that complete anonymity of the respondents was verified. As a result of this procedure, there is no possibility of de-identifying or de-anonymizing the records prior to analysis.

Implicit informed consent is given by participating after being fully informed of the objectives of the study. Written or verbal informed consent is not obtained during this study for two reasons. First, recording information that could be used to identify the participants (especially names and e-mail-addresses but also IP-addresses, time of participation) is explicitly prohibited by the data security officer. Therefore, written or verbal consent could not be obtained. Second, recording these data would have lowered the trust of the respondents in the anonymity of the study and would have foiled the logic of the RRT survey. The study was performed according to the principles of the Helsinki declaration.

## Sample

The survey was carried out in the catchment area of the SBP, the umbrella organization for organised sport in the Palatinate. Access to recreational and amateur athletes was achieved with the cooperation of the SBP via different media (such as press releases and reports in regional newspapers) and using a snowball process. The sports-practicing public was notified about the survey via the Sports Association, with the organization and the group of researchers writing several times (in “special newsletters” to all subscribers, approximately 8,000 people, and in emails to the addresses held by the SBP for association officials, trainers, participants in events, etc., approximately 9,900 people). In all of the communications, the persons contacted were first asked to take part in the survey themselves. They were additionally asked to further distribute the information about the survey within their club, their training group, their circle of friends, etc.

Due to the snowball process, the transmission of information depended on a large number of factors that are almost impossible to influence, e.g., the commitment of those individuals initially contacted during the distribution, the (communication) networks in the clubs, the intensity and frequency of information exchange, etc. This gave rise to biased distributions with regard to the types of sport practiced (see Table 1). A further reason for this bias was the fact that, in the questionnaire, non-team sports were mentioned, while the distribution of athletes in the SBP was determined by the distribution of members across the constituent specialist clubs.

**Table 1:** Distribution of responses by types of sport practiced compared with the distribution of sports practice in the SB Pfalz. N = 1,620 data sets stating the type of sport practiced in the sports with the highest priority.

Sport	Number of respondents	Percentage of respondents	Percentage of athletes in SBP
Football	297	15,2	30,0
Running *	197	10,1	-
Athletics *	183	9,4	3,9
Tennis	137	7,0	6,3
Handball	100	5,1	3,5
Aquatics	91	4,7	1,7
Gymnastics *	83	4,3	18,9
Volleyball	69	3,5	1,0
Cycling	68	3,5	1,0
Triathlon	64	3,3	0,2
Badminton	60	3,1	0,9
Table tennis	58	3,0	2,9
Dancing	49	2,5	1,2
Judo	48	2,5	0,7
Shooting	37	1,9	3,8
Alpine Skiing	29	1,5	2,1
Equestrian	28	1,4	2,1
Bowling	22	1,1	0,5

* the asterisk denotes a sport with special characteristics that is explained in the text.

Two oddities in Table 1 (indicated by *) were influenced by the special structure of sports in Germany and do not reflect sampling errors. First, a rather small number of respondents stated “gymnastics” as their type of sport. The *Palatinate Gymnastics Association* covers different sport disciplines and sport clubs that are historically under the umbrella of German “Turnen”. Additionally, this association covers all types of sport that lack a special regional umbrella organisation. Therefore, the number of members of the *Palatinate Gymnastics Association* far exceeded the share of respondents practicing gymnastics. The opposite phenomenon explained the difference between the number of athletes involved in “jogging/running” and in "athletics" and the number of members of the *Palatinate Athletics Association*. Running competitions are governed by the Athletics Association and there is no special runners' association. Nevertheless, it is possible for athletes who do not belong to any athletic club to take part in competitions, such as city marathons or fun runs, which can be organized to allow runners who do not belong to a sports club to participate (that is, these competitions do not require athletes to have a starting license).

Our dataset comprised 1,930 responses containing individual data and 1,964 responses containing questions about types of sport (here, one person can contribute with two datasets about their sports behaviour). The first question asked for the participant’s zip code to assure that the person lives in the region of Palatinate. Then we asked if the person is or had been formerly engaged in sports. For the RRT questions we prioritise one sport (see methods section) stated by the respondent.

It was not easy to quantify the response rate due to the snowball process. We only can estimate the rate from those persons who directly received the questionnaire. We scored all newsletter subscribers from SBP, a sample with more than 10,000 persons, with a special newsletter. Additionally, an email went out to all instructors and participants of an advanced training course (about 9,000). There was some overlap between these two groups, so that 10,000 direct mail recipients would be a conservative estimation. Using this number, we had a response rate of less than 20%. Because of other processes, like information sharing across sports clubs and the several newspaper articles about the survey, the response rate was clearly lower. 65.8% of the responses came from male athletes and 33.42% from female (the gap between male and female respondents comes from item non-responders). This distribution did not correspond to the population distribution in Rhineland-Palatinate, but it was very close to the distribution of sexes at the SBP and, thus, presumably to that of the population of the Palatinate who actively practice sport and who are the targets of the study.

Furthermore, the sample was characterized by high educational and professional status, which was evident from the high proportion of persons having completed secondary education and the that almost one third of respondents had a university degree.

## Results

### Results–prevalence of doping

**Fig 3:**
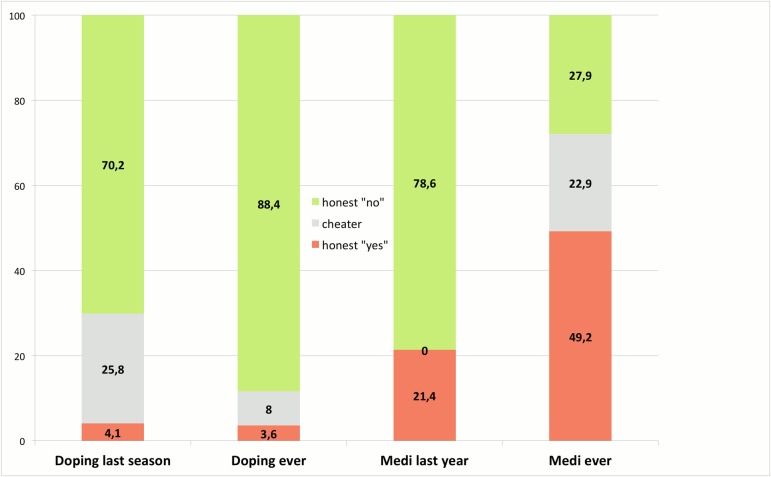
Results of the RRT questions for doping and sport-induced self-medication (medi).

When assessing the prevalence of substance use, we estimated the rate of honest yes respondents and of honest no respondents directly from the data. Typically, both shares did not sum up to 100%, as there remains a range of indifference because some participants (deliberately or by chance) did not obey the RRT instructions correctly (“cheaters” in the RRT-terminology). Therefore, we calculated an interval for the true prevalence: The lower boundary is the proportion of honest yes respondents (the red bars in Fig 3), while the upper boundary is defined by the proportion of honest no respondents (the green bars in Fig 3). The breadth of the interval between these two shares refers to the level of indifference. It ranges from the estimated proportion of honest yes respondents to 100% minus the share of honest no respondents.

### Results–prevalence for sport-induced self-medication

Regarding whether athletes in the SBP deliberately used prohibited substances and/or methods with the aim of enhancing their competitive performance during the last season of their named sport, the proportion of honest “yes” respondents was 4.29%. With a proportion of honest no responders of 69.52%, the true prevalence falls between 4.29% and 30.48%. The percentage of respondents who doped at any point in their sports life to date falls between 3.35% and 10.55%. The proportion of honest no respondents was 89.45%. The much larger range of the interval due to non-compliance to the RRT instructions for the first question may be surprising at first, but it was similar to that seen in comparable studies of deviant behaviour [30–31]. A possible explanation for the fact that non-compliance to RRT-instructions on the question relating to the last season is seen more often than on the question relating to one’s entire sporting life might lie in the proximity of the response to the embarrassing question to the deviant behaviour itself. The risk of being identified is higher for deviance that occurred recently than it is for deviance that occurred at some point during a long period of time.

Another question asked whether the athletes in the SBP used any substance for sport-induced self-medication without intending to enhance their competitive performance in the past year. With regard to substance use in the last year, the proportion of honest “yes” respondents in the last year was 21.40%. The proportion of honest no responders was estimated at 78.60%. Thus we did not have an interval for this question. With regard to the lifetime prevalence of substance use for self-medication in a sport-related context, we obtained an estimate of 49.20% honest yes respondents and 27.87% honest no respondents. The upper limit of the interval for lifetime use is 72.1%.

### Hypotheses Tests

The results broken down by gender are shown in Tables A and B in S1 Appendix. The differences between male and female athletes are highest for the question regarding whether they have ever taken prohibited substances (doping), but the differences between genders did not reach significance for any question. The cell frequencies for the questions question were very low, so each estimate to compare sexes is relatively unreliable.

For the social status as well as the sport involvement hypotheses, the cell frequencies were even lower, which meant that this study did not provide any evidence either to support or to reject these hypotheses.

Without a pre-defined hypothesis, we additionally compared the substance use for doping purposes to the use of medication in the sport context for other purposes than to enhance performance. We have N = 616 respondents who answered both the doping question and the question for sport-induced self-medication during the last season or year and N = 786 answers for both questions about lifetime use. Although numerically different, the prevalence for doping and medication use did not differ significantly for the last season/last year question (see supporting information Table C in S1 Appendix). Likewise, there is a significant difference in these questions on the respondent’s lifetime in both the number of honest “yes” and the number of honest “no” responses (see Table D in S1 Appendix).

## Discussion

We found a last season prevalence of doping over 4% and a lower lifetime prevalence (3.6%). The common logic would say that lifetime prevalence can only be the same or higher than the last season's prevalence. So these results may puzzle at first and need more explanation. First, these results are estimations that contain a certain error term. Because of the result of the significance test we can say, that the difference isn’t a relevant difference. Second, different arguments affect the RRT procedure. For each RRT question we have to use a randomization process to divide the sample into 2 groups. This means that between the two questions (last year and lifetime) the sample that has to answer honestly can be assembled in a different way. For example, in the first question respondents get the instruction to answer honestly and in the second to answer always yes (respectively to answer the right or left question). The information on the prevalence only can come from those who answer honestly, so that the randomization process can lead to a different estimation.

When interpreting our results in a public health context, we must conclude that in spite of the low prevalence rate of doping, from a population of (conservatively estimated) more than 20 million amateur and recreational athletes in Germany, nearly 900,000 are estimated to have used illegal substances in the last season to enhance their sporting performance. In addition, more than four million amateur and recreational athletes have used pharmaceuticals for purposes other than performance enhancement in the context of sport. Therefore, we must concede that substance use without the aim of performance enhancement in sports-related contexts is a larger public health problem than doping (substance use with the aim of performance enhancement), but that both problems indeed affect large numbers of individuals. These forms of sport-induced use of medication at least partly counteract health implications, which are often seen as a side effect of recreational sport. In contrast to the cited literature we could not find a gender difference in such a way that substance use is a male problem. Maybe the multidisciplinary sample and the recreational sports level can cause this. We found the reported differences especially at students and bodybuilding studies.

Regarding the RRT questions about prohibited substances that are used with the aim of enhancing performance, we highlight the tension between the fact that, at least in principle, the WADC applies to recreational and amateur athletes and the fact that we cannot assume that casual athletes are aware of the list of banned substances and its contents. Responses to our survey are thus biased by this tension in different ways. On the one hand, we had respondents who take substances that they regard as prohibited but that are not prohibited according to the WADC, leading to an overestimation of the prevalence of doping. On the other hand, we had respondents who take substances that they believe are permitted but are actually prohibited according to the WADC, leading to an underestimation of the prevalence of doping. Nevertheless, the blurred line that separates conforming behaviour from deviance in amateur and recreational sport is a strong argument for a tailored Anti-Doping regime in this realm to preserve fairness and the spirit of sport [57].

At this point, we lack information about how familiar recreational and amateur athletes are with the WADA list of banned substances, and thus which substances they consider to be prohibited or performance enhancing. Further studies are required on this topic so that this bias can be factored into future models. In this study we are measuring substance use, as we are focused on how many athletes reported taking substances they considered to be performance enhancing and prohibited.

When comparing the rates of substance use for sport-induced self-medication with rates of doping, we show that motives other than performance enhancement are the far more prevalent phenomenon. One argument for testing in or outside of competitions is that anti-doping measures protect athletes' health. It is therefore interesting that we find the more widespread behaviour is related to medication, which is not under the purview of competitions, federations, or sports organisations. In our opinion, there is no way to introduce a testing system similar to elite sports in this domain, and any type of organized regulation is destined to fail in this area. One reason is because there is no money to do it. Another reason is that recreational athletes use substances as they see fit if they are for medication purposes (but possibly prohibited in the sports environment). There are lots of areas in amateur sports that cannot be controlled by sports organizations (no license need, private sports participation, fitness studios etc.). If we look at the reported motivations for substance use, performance enhancement has less of an effect than do other motivations like pain reduction, recuperation, or mood improvement. Additionally, prohibited substances are less used than permitted substances.

## Limitations of the Study

This study has limitations, so generalizations from the results should be made with caution.

First of all, the use of RRT aims to address the problem of social desirability bias, although this limits the reliability of any attempts to measure embarrassing properties by principle. When estimating the proportions of the three response types (honest yes respondents, honest no respondents, and cheaters in RRT-terminology) it is apparent, as has already been shown in other studies, that the range of indifference due to respondents not obeying the RRT instructions correctly is lower when the embarrassing question (here, doping) asks about a broader and more distant period of time (here, any point in one’s athletic life). When the behaviour takes place more recently, the proportion of indifference is higher. This finding is consistent with findings from work on elite German athletes [30–31] and on substance use by students [13]. It strongly supports the assumption that this range of indifference is largely influenced by deliberate cheating while random fluctuations in respondents’ behaviour adds little to this proportion. The sensitivity of the proportion of cheaters to the temporal proximity of the doping behaviour, and the degree of threat from confessing to the embarrassing characteristic, is a strong argument for using RRT even in recreational and amateur sport where the social desirability bias is plausibly lower than in elite sport. This means that with the use of the RRT method we are sure to reduce this general limitation problem. Additionally, we can show that with the RRT the proportion of social desirable answers are lower and we can measure this proportion.

Second, the studied sample was not necessarily representative of all amateur athletes or of all sports represented by the SBP, due to the rather poor response rate in comparison to the athletes taking part in different sports (see Table 1). Nevertheless, it provided the first estimate of the prevalence of substance use in a population survey using a technique that elicits reliable results. These results are comparable to other studies in recreational sport, as well as to studies on doping in elite sports. Nevertheless, the results should be interpreted cautiously until they can be independently replicated.

Third, the athletes in our sample may not have known which products are banned and which are not in their particular sport. We made a distinction between doping and medication for other purposes than performance enhancement in the analysis. However, though the respondents were asked if they had “ever used prohibited substances or methods with the aim to enhance their performance” or “ever used substances with other aims than performance enhancement”, we did not know if this distinction was clear for the individual respondents or if the substances they believed to be banned matched those actually banned by the WADC. Therefore, the results should be understood as reflecting the relative frequency of an openness to transgress between substance categories (i.e., from acceptable to prohibited) rather than as information on the frequency of genuine anti-doping rule infringements. Additionally, we cannot distinguish between substance use that is a kind of self-medication and those used under the guidance of a physician. We only asked about intent and not the kind of medication.

Finally, due to the challenges of implementing no-cheater detection for RRT [54], the setup may not have been not fully optimized to reduce error variance in the estimators. On the other hand, given the intrinsic difficulties in obtaining reliable prevalence data for sensitive behaviour and the paucity of doping prevalence data in the general population, our study had the distinction of being the first to report results in amateur athletes in a specific region across all sports using a method that allows us to reduce bias in surveys on sensitive issues.

## Supporting Information

S1 Appendix Explanation to the Bootstrap-tests for significance and Tables A-D with the different comparisons (with the independent variable “sex” and between the questions for doping and medi). (DOCX)
