# Supplementary material for: Sport-Induced Substance Use—An Empirical Study to the Extent within a German Sports Association
Source: PLoS One. 2016 Oct 26;11(10):e0165103. doi: 10.1371/journal.pone.0165103 (PMC5082616; doi:10.1371/journal.pone.0165103)
Supplement: S1 Appendix — (DOCX) [file pone.0165103.s001.docx]

**S1 Supporting Information**

**Significance Tests**

*Explanation to the Bootstrap-tests for significance:*

In the second and third column of the tables, the raw values are reported. Column four contains the differences between these values. In column five the reference bcα- (bias corrected and accelerated) value is reported. If for positive (negative) values of the difference, the limit is also positive (negative), then this difference is significant at the given significance level (p=0.05).

**Table A. Comparison of the question doping last season with independent variable “sex” (N=655).**

|  | **Male** | **female** | **Diff** | **bcα-value** |
| --- | --- | --- | --- | --- |
| **honest yes** | 0.0135 | 0.0954 | 0.0819 | -0.0405 |
| **Cheater** | 0.1964 | 0.1251 | 0.1672 | -0.2465 |
| **honest no** | 0.7902 | 0.2751 | -0.2491 | 0.2583 |

**Table B. Comparison of the question doping ever with independent variable “sex” (N=824).**

|  | **male** | **female** | **Diff** | **bcα-value** |
| --- | --- | --- | --- | --- |
| **honest yes** | 0.0238 | 0.0346 | 0.0109 | -0.0708 |
| **Cheater** | 0.0477 | 0.0660 | 0.0183 | -0.2288 |
| **honest no** | 0.9286 | 0.8994 | -0.0292 | 0.2937 |

**Table C. Comparison of the questions doping last season and for sport-induced self-medication (medi) last year (N=616).**

|  | **Doping** | **Medi** | **Diff** | **bcα-value** |
| --- | --- | --- | --- | --- |
| **honest yes** | 0.0325 | 0.2681 | 0.2356 | -0.1346 |
| **Cheater** | 0.2181 | 0.0000 | -0.2180 | 0.0000 |
| **honest no** | 0.7495 | 0.7319 | -0.0176 | 0.3298 |

**Table D. Comparison of the questions doping and for sport-induced self-medication (medi) ever (lifetime prevalence, N=786).**

|  | **Doping** | **Medi** | **Diff** | **bcα-value** |
| --- | --- | --- | --- | --- |
| **honest yes** | 0.0390 | 0.4917 | 0.4527 | 0.3548 |
| **Cheater** | 0.0978 | 0.1328 | 0.0351 | -0.2361 |
| **honest no** | 0.8633 | 0.3755 | -0.4878 | -0.1370 |
